# Analysis of the Anti-Inflammatory and Analgesic Mechanism of Shiyifang Vinum Based on Network Pharmacology

**DOI:** 10.1155/2021/8871276

**Published:** 2021-01-13

**Authors:** Hui Tian, Linli Wei, Yunxiu Yao, Zhaoqing Zeng, Xue Liang, Hua Zhu

**Affiliations:** ^1^Guangxi University for Nationalities, Nanning 530006, China; ^2^Guangxi University of Chinese Medicine, Nanning 530200, China

## Abstract

**Objective:**

The possible core active compounds and potential mechanism of action of Shiyifang Vinum were explored through network pharmacology and *in vitro* enzyme activity verification experiments.

**Methods:**

We screened the core active components and the action targets of Shiyifang Vinum through the TCMSP database and literature mining and drew a Venn map of the intersection with anti-inflammatory and analgesic-related gene targets. Go and KEGG analyses were enriched with the David database. The compound target pathway network was constructed using Cytoscape 3.6.1. The binding strength of core active compounds and target proteins was verified through molecular docking, and the direct effects of Shiyifang Vinum and four monomer compounds on COX-2 enzyme activity were detected through an *in vitro* enzyme activity test.

**Results:**

14 active compounds and 11 targets were screened out from Shiyifang Vinum through TCMSP database and literature mining; 252 GO entries were obtained by GO analysis, and 114 signal pathways were screened by KEGG analysis. The results of the molecular docking showed that the core compounds and target proteins had strong binding activity. *In vitro* validation experiments showed that both the Shiyifang Vinum and the four monomer compounds could inhibit the activity of COX-2.

**Conclusion:**

This study preliminarily explored the potential active compounds and target proteins of the anti-inflammatory and analgesic effects of Shiyifang Vinum, which could provide a scientific basis for further study on the anti-inflammatory and analgesic mechanism and material basis of this recipe.

## 1. Introduction

Shiyifang Vinum is a secret recipe presented by Xi'en Liang, a famous orthopaedics specialist in Guangxi. It is a traditional Chinese medicine preparation composed of 13 herbs, including rhubarb, safflower, Lycopus, lignum sappan, Paris polyphylla, ground beetle, amber, dragon's blood, notoginseng, Pyritum, dipsacus, nux vomica, and myrrh. It has the effect of relieving pain and swelling and dispersing blood stasis and is used for all kinds of injuries, falls, bruises, redness, and swelling. The prescription and main chemical components are shown in Table 1. This medicine is widely used in the treatment of knee osteoarthritis, frozen shoulder, and fracture, among others, with definite curative effects and positive effects on inflammatory pain [1–3]. However, there are no reports in the literature of the material basis, anti-inflammatory mechanism, and analgesic effect of Shiyifang Vinum. Therefore, it is necessary to explore these items using network pharmacology.

In 2007, British pharmacologist Hopkins [4] put forward the concept of “network pharmacology” for the first time in Nature Biotechnology. The concept of network pharmacology was accepted and widely used in the field of traditional Chinese medicine soon after it was proposed. Its overall advantages provide new ideas for the study of complex traditional Chinese medicine system. On the basis of systems biology and bioinformatics, it constructs the interaction network of compounds, targets, pathways, and diseases, systematically observes the multichannel regulation of drugs on the disease network, and analyses and predicts the mechanism of drug action. However, the results of pure computer simulation analysis are often questioned, so it is necessary to provide the basis for the accuracy of network pharmacological analysis results through corresponding experiments [5]. Network pharmacology integrates the technology and knowledge of systems biology, multidirectional pharmacology, bioinformatics, and computer science. It has the characteristics of integrity and systematicness. It can be used to reveal the complex biological network relationship among drugs, ingredients, targets, and diseases so as to improve the efficiency of new drug research and development. It is especially suitable for the multicomponent and multitarget collaboration of traditional Chinese medicine using biological network research. The application of network pharmacology in the research of traditional Chinese medicine mainly focuses on the following 4 aspects: (1) explaining the mechanism of multicomponents and multitargets of Chinese medicine; (2) selecting active components of Chinese medicine; (3) new use of old drugs (drug reorientation); (4) explaining the relationship between principal components and compatibility of Chinese herbal medicine [6]. At present, many people have used network pharmacology to study the mechanism of action of traditional Chinese medicine, and the results have been confirmed. Fan et al. used network pharmacology to study the mechanism of Gegen Qinlian Decoction on colorectal cancer. A total of 118 active chemical components, 20 key targets, and 6 pathways were screened out, which provided a research basis for further research on the mechanism of Gegen Qinlian Decoction on colorectal cancer [7]. Zhu et al. elucidated the mechanism of Hugan Buzure Granule in the treatment of liver fibrosis by network pharmacology, predicted 25 active components, 115 key targets, and 127 related signal pathways of Hugan Buzure Granule, revealing the characteristics of multicomponent, multitarget, and multichannel of Hugan Bupi granule in the treatment of liver fibrosis [8]. Ou et al. used the methods of network pharmacology and molecular docking to elucidate a variety of potential mechanisms of Qing Guang An Granule in the treatment of glaucoma. The results suggest that Qing Guang An Granule may play a protective role by acting on retinal ganglion cells and optic nerve at the molecular level [9]. Traditional Chinese medicine compound preparation has the characteristics of complex chemical composition, multitarget, multilevel and synergistic effect, which is consistent with the holistic and systematic characteristics of network pharmacology [10]. It can be seen that the method of compound Chinese medicine network is feasible. In this study, we screened out the potential core components of Shiyifang Vinum through network pharmacology and predicted the material basis, potential targets, and action mechanism of its anti-inflammatory and analgesic effects so as to provide a reference for the later study of the mechanism of action and Q-maker.

## 2. Methods

### 2.1. Screening of Potential Pharmacological Active Ingredients and Targets of Shiyifang Vinum

Thirteen kinds of traditional Chinese medicine, such as Sappan Lignum and safflower, were used as keywords to search for all the chemical components in the TCMSP database. Seven were not included in the TCMSP database, but we found the chemical constituents of these seven herbs after searching the literature. Among them, some chemical constituents of lignum sappan [11], Paris polyphylla [12], ground beetle [13], and dragon's blood [14] were retrieved from TCMSP. The target proteins of the chemical components in Shiyifang Vinum were identified by selecting the target prediction function in the TCMSP database as the screening conditions of active compounds with oral bioavailability (OB) ≥ 30% and drug-like compounds ≥0.18 [15]. The corresponding genes of all target proteins were obtained with the help of the UniProt database.

### 2.2. Screening of Anti-Inflammatory and Analgesic Targets of Shiyifang Vinum

The disease-related genes were collected from the Genecards database, and the related anti-inflammatory and analgesic targets were obtained by entering the keywords “anti-inflammation” and “analgesic.” Potential targets were obtained by intersecting the component targets and anti-inflammatory and analgesic targets of Shiyifang Vinum. A Venn map was drawn on the Venny 2.1.0 website.

### 2.3. Construction of a PPI Network for Targets of Shiyifang Vinum

The cross-gene interaction files of the components and anti-inflammatory and analgesic effects were downloaded from the string database, and the PPI network was drawn with Cytoscape 3.6.1 software.

### 2.4. Go and Kyoto Encyclopedia of Genes and Genomes (KEGG) Analysis of Shiyifang Vinum

The key targets of the anti-inflammatory and analgesic effects of Shiyifang Vinum Obtained were imported into the David database. Go biological process enrichment analysis and KEGG signal pathway enrichment analysis were selected to draw a bubble diagram with the Omicshare database, and a histogram was drawn with GraphPad Prism 6.0 software.

### 2.5. Construction of the Active Ingredients-Targets-Pathways Network of Shiyifang Vinum

Active ingredient, target, and pathway data were imported into the Cytoscape 3.6.1 software to construct the active ingredients-targets-pathways network. Node degree value was set to more than two times the median of all nodes of connectivity as the standard to screen, key nodes were determined, and the core active ingredients of Shiyifang Vinum were explored.

### 2.6. Active Ingredients-Targets Molecular Docking of Shiyifang Vinum

The 3D-structure pdb format file of the target protein was downloaded from the PDB database. Ligand and nonprotein molecules in the target protein were removed by discovery studio 2020 client software and then saved as a pdb file. The 2D-structure file of the core compound was downloaded from the PubChem database. The protein file after water hydrogenation was uploaded to pdbqt format file through the Py Rx software, and then the compound file was uploaded to minimise its energy. It was transformed into a pdbqt format file. Finally, Vina was used for molecular docking.

### 2.7. *In Vitro* Validation Experiment

#### 2.7.1. Instruments and Reagents

The full-wave multifunctional enzyme-labelling instrument (Tecan (Shanghai) Trading Co., Ltd.); micropipette (Eppendorf AG); ultrapure water analyser (model: millibo direct Q-5); vortex instrument (Shanghai Luxi brand instrument company); DMSO (Batch no.1121e037, Beijing Solebao Technology Co., Ltd.); freeze dryer (model: Christ alpha) 1-2 ldplus); 1/100000 scale (Sedolis Scientific Instruments Co., Ltd.); 96-well blackboards (Batch No. 15419010, Corning Co., Ltd.); centrifuge tube; and COX-2 Inhibitor Screening Kit were purchased from Beyotime Biotechnology (Shanghai, China); the Shiyifang Vinum was provided by the First Affiliated Hospital of Guangxi University of traditional Chinese medicine; the quercetin and kaempferol were purchased from the National Institutes for Food and Drug Control (Beijing, China); the luteolin and apigenin were purchased from Shanghai Yuanye Bio-Technology Co., Ltd. (Chengdu, China).

#### 2.7.2. Sample Preparation and Experimental Operation Method

Measure the appropriate amount of Shiyifang Vinum, place it in a water bath pot at 50°C, and dry it in a freeze dryer. Accurately weigh the appropriate amount of quercetin, luteolin, apigenin, and kaempferol and the dried products of Shiyifang Vinum, add appropriate DMSO to dissolve, prepare the mother liquor of 1 mg/ml, apply ultrasonic treatment for 30 min and 12000 r/min centrifugation for 10 min, pass the supernatant through the membrane for standby, and configure it into appropriate concentration gradient for use.

The COX-2 inhibitory activities of four monomer compounds and Shiyifang Vinum were detected with the COX-2 inhibitor screening kit. The blank control, 100% enzyme activity control, positive inhibition control, and sample wells were prepared, with three repeat wells in each group. With this kit, fluorescence was determined using an enzyme-labelled instrument, with an excitation wavelength (Ex) of 560 nm and an emission wavelength (EM) of 590 nm [16]. The inhibition rate of each sample was also calculated.

(1) $$\begin{array}{c} \text{Inhibition activity }\left(\text{\%}\right)=\frac{\left(A_{1}-{\text{A}}_{2}\right)}{\left(A_{1}-A_{3}\right)}\times 100\text{\%,} \end{array}$$

where *A*_1_, *A*_2_, *A*_3_ are the fluorescence value of 100% enzyme activity control group, sample group, and blank control group, respectively.

The concentration values of each sample and celecoxib were taken as the *x*-axis, the COX-2 inhibition rate of each sample and celecoxib inhibitor were taken as the *y*-axis, and the IC50 of 50% inhibition concentration was calculated with probability unit regression (probit) SPSS 20.0 software.

## 3. Results

### 3.1. Screening of Potential Pharmacological Active Ingredients and Targets of Shiyifang Vinum

We explored the potential targets of the 104 potential pharmacologically active ingredients by mining TCMSP databases, which yielded 414 targets.

### 3.2. Screening of Anti-Inflammatory and Analgesic Targets of Shiyifang Vinum

**Figure 1:**
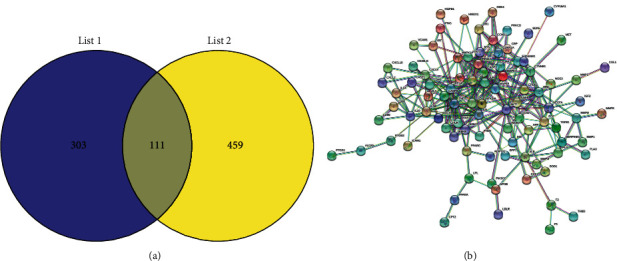
(a) Venn diagram of the intersection of the Shiyifang Vinum gene and the anti-inflammation and analgesia genes. (b) PPI network of targets of Shiyifang Vinum.

A total of 570 anti-inflammatory and analgesic-related targets were screened from the Genecards database, and the component targets and anti-inflammatory and analgesic disease targets were then graphed in a Venn diagram. Also, 111 overlapping targets were obtained, as shown in Figure 1(a), which are the related targets of active components of traditional Chinese medicine acting on diseases.

### 3.3. Construction of a PPI Network for Targets of Shiyifang Vinum

A total of 111 common targets were input on the string online data platform, and the PPI network was obtained after analysis. Each node represents a protein, and the connection between nodes represents the interaction between two proteins. Different colors depict different types of interaction. More lines indicate a greater correlation and it can be seen in Figure 1(b) that the interaction between targets of Shiyifang Vinum is a huge interactive network rather than a single one.

### 3.4. Go and KEGG Analyses of Shiyifang Vinum

**Figure 2:**
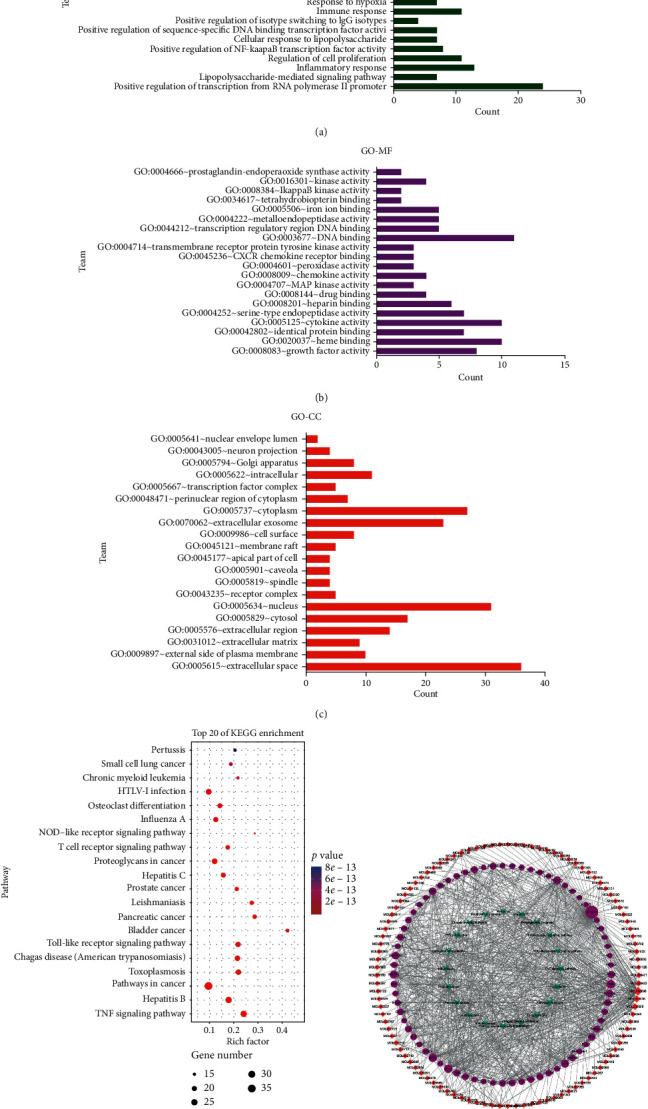
(a) Results of the Go biological process of Shiyifang Vinum. (b) Results of the Go molecular function of Shiyifang Vinum. (c)Results of the Go cellular component of Shiyifang Vinum. (d) Results of the KEGG enrichment of Shiyifang Vinum. (e) Active ingredients-targets-pathways network of Shiyifang Vinum.

There were 252 Go entries in David (*P* < 0.05), including 193 biological processes (BP), 26 cell compositions, and 33 molecular functions (MF). Biological processes are mainly enriched with the transcription of RNA polymerase II promoter, inflammatory response, immune response, cell proliferation, and other processes; MF are mainly concentrated in DNA binding, cytokine activity, heme binding, and other processes; and cell composition is mainly concentrated in the extracellular space, nucleus, and cytoplasm, among other organelles. The KEGG pathway was enriched and screened to obtain 114 signal pathways (*P* < 0.05). Among them, the significant results of the first 20 pathways were drawn into a bubble diagram. The key targets of Shiyifang Vinum were mainly concentrated in the cancer pathway, Hepatitis B and TNF signalling pathways, the HTLV-1 infection and toll-like receptor signalling pathways, the T-cell receptor signalling pathway, and the osteoclast differentiation and nod-like receptor signalling pathways, suggesting that they play an important role in the anti-inflammatory and analgesic effects of Shiyifang Vinum (Figures 2(a)–2(d)).

### 3.5. Construction of Active Ingredients-Targets-Pathways Network of Shiyifang Vinum

The active ingredients-targets-pathways network consists of 196 nodes (104 compound nodes, 72 target nodes, and 20 pathway nodes) and 1005 edges. Red represents active compounds, purple represents targets, and blue represents pathways. Based on the degree of compound target pathway network, quercetin, luteolin, apigenin, kaempferol, and *β*-sitosterol ranked among the top five in the network, and the degree values were 56, 32, 22, 20, and 17, respectively. PTGS2, TNF, AKT1, RELA, and PIK3CG were the top five targets, with degree values of 105, 45, 41, 41, and 38. These results are an indication that these compounds and targets may be the main anti-inflammatory and analgesic compounds and targets of Shiyifang Vinum. We predict that these compounds and targets will play a major role in the activity of Shiyifang Vinum. (Figure 2(e))

### 3.6. Compound Target Molecular Docking of Shiyifang Vinum

Molecular docking technology was used to carry out systematic docking to further study the binding ability of core active compounds with key targets. It is generally accepted that the binding energy is less than 0, which indicates that the compound can spontaneously bind to the protein. The more stable the conformational stability of the compound and protein binding, the lower the energy and the greater the possibility of interaction [17]. In some studies, the binding energy of ≤ −5.0 kJ/mol was used as the screening standard [18, 19]. In this study, 14 core active compounds with a moderate value greater than the average value in the compound target pathway network of Shiyifang Vinum were docked with 11 target proteins. The results show that except for ESR1, NFKBIA, and RELA, there were no relevant data for the other 8 target proteins and 14 cores of Shiyifang Vinum. The binding energies of the compounds are far less than −5.0 kJ/mol, as shown in Table 2. Therefore, the core compounds and target proteins have good binding ability, which indirectly proves that the results of predicting network pharmacology are reliable and accurate. All of the compounds interacted with the selected targets, respectively, and interacted with two or more targets simultaneously, which indicated that Shiyifang Vinum could play a positive role in the multitarget and multichannel advantages of traditional Chinese medicine. These results preliminarily explained the multitarget mechanism of the anti-inflammatory and analgesic effects of Shiyifang Vinum.

### 3.7. Sample Preparation and Experimental Operation Method

**Figure 3:**
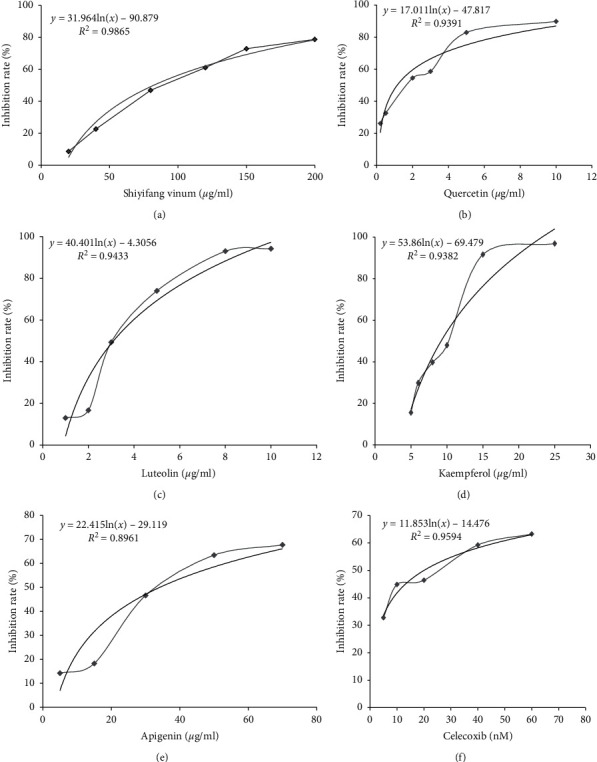
Dose-response curves for inhibition of COX-2.

COX-2 was selected as the research target for this study according to the research results of network pharmacology, and the inhibition activity of COX-2 was verified using Shiyifang Vinum, and quercetin, luteolin, apigenin, kaempferol, and other monomer compounds. The dose-response curve for the verification experiment is shown in Figure 3. The results show that Shiyifang Vinum and four monomer compounds have a significant inhibitory effect on COX-2, and the IC_50_ of quercetin, luteolin, kaempferol, apigenin, and Shiyifang Vinum was 1.198 *μ*g/ml, 3.102 *μ*g/ml, 8.774 *μ*g/ml, 35.518 *μ*g/ml, and 106.95 *μ*g/ml, respectively. The IC_50_ of celecoxib, a selective COX-2 inhibitor, was 20 nM, which was consistent with the results reported in the kit (IC_50_ was approximately 10–100 nM) and the test results (17.79 nM) are reported in the literature [19].

## 4. Discussion

In the part of network pharmacology research, the components of Pyritum and amber were not included in tcmsp database. In order to unify the standard, no other database was searched. No other database was searched in order to unify the standard. However, according to the literature review, Pyritum has the effects of dispersing blood stasis and removing pus, relieving pain, calming convulsions, and extending tendon and bone grafting. It may promote the differentiation of bone marrow mesenchymal stem cells into osteoblasts and the growth of bone callus by increasing ATF4 expression [20]. Amber has the effects of calming the nerves, promoting blood circulation, diuresis, and so on. It is mainly used in the auxiliary treatment of convulsions, epilepsy, palpitations, and insomnia and is also commonly used in traumatic injuries, carbuncle, gangrene, etc. [21].

We constructed the compound target pathway network of the global anti-inflammatory and analgesic effects through the network pharmacology method and found that the anti-inflammatory and analgesic mechanism of Shiyifang Vinum was to regulate multiple targets and multiple pathways to intervene and work together. Based on network pharmacology data, quercetin, luteolin, apigenin, kaempferol, *β*-sitosterol, and other compounds are the most important nodes of the entire network. Previous studies have shown that quercetin, luteolin, apigenin, kaempferol, and *β*-sitosterol, among others, have obvious anti-inflammatory and analgesic effects [22–29], which can explain to a certain extent that the network pharmacology method has feasibility. PTGS2, TNF, AKT1, RELA, PIK3CG, and other targets interacted with the active components of Shiyifang Vinum, and the degree value was higher. PTGS2, also known as COX-2, can be inhibited by inhibitors, thus blocking the transformation of arachidonic acid into prostaglandins, reducing the content of prostaglandins, and alleviating inflammation and pain symptoms of tissues involved in prostaglandins [30]. TNF is a proinflammatory factor; TNF-*α* can mediate inflammatory reaction, induce the production of IL-1 *β*, IL-6, and other inflammatory factors, and aggravate the local inflammatory reaction [31]. Rela is a member of the NF-*κ* B family. Its posttranslational modification can precisely regulate the transcriptional activity of NF-*κ* B and plays an important role in regulating inflammation, cell proliferation, survival, early viral response, cytokine secretion, and proinflammatory effect [32]. PIK3CG is a key regulator of inflammation and oxidation and involves many inflammatory diseases. Studies have found that the treatment of PIK3CG deficiency in the immune system of mice with specific PIK3CG inhibitors can inhibit the inflammatory process in the vascular wall [33]. AKT1, also known as protein kinase B *α*, can reduce the inflammatory response induced by angiotensin II in human umbilical vein endothelial cells by inhibiting AKT expression [34]. Therefore, they may be potential targets of the anti-inflammatory and analgesic effects of Shiyifang Vinum.

Based on the enrichment of the KEGG pathway, we found that the key targets of the anti-inflammatory and analgesic effects of Shiyifang Vinum involve TNF signalling pathway, toll-like receptor signalling pathway, and T-cell receptor signalling pathway, which mainly regulate the inflammatory reaction and apoptosis-related processes. Furthermore, molecular docking technology was used to verify the core active components and target proteins on the prediction results of network pharmacology. The verification results show that the core active compounds and target proteins have strong binding ability. It can also be seen that these core compounds have high binding activity with receptor proteins. It is speculated that the anti-inflammatory and analgesic mechanism of Shiyifang Vinum may be the core compound, and target protein binding plays an anti-inflammatory and analgesic effect. The rationality of the web-based pharmacology database is limited by its completeness and rationality.

COX-2 is closely related to the occurrence and development of inflammation. It is a key enzyme that catalyses anachronic acid into prostaglandins that mediate pain and inflammation [35]. In this study, an *in vitro* enzyme activity test was used to detect the direct effect of Shiyifang Vinum and four monomer compounds on COX-2 enzyme activity. The reliability of the method was verified with the positive drug celecoxib. It was found that Shiyifang Vinum and four monomer compounds had different degrees of inhibition on COX-2, and the inhibitory activity of COX-2 showed a significant dose-dependent relationship. These results also showed that the inhibitory activity of the monomers on COX-2 was quercetin > luteolin > kaempferol > apigenin > Shiyifang Vinum. It has been reported that quercetin, luteolin, apigenin, and kaempferol can inhibit COX-2 expression, which also indicates that quercetin, luteolin, apigenin, and kaempferol can significantly affect COX-2, and also preliminarily prove that the research results related to network pharmacology are scientific and reliable [36–39].

In conclusion, based on network pharmacology, this study explored the core active components, key targets, and important signalling pathways of the anti-inflammatory and analgesic effects of Shiyifang Vinum and verified the main active components and targets through molecular docking. At the same time, the enzyme activity of the COX-2 target was tested *in vitro*, which verified the reliability of the network pharmacology analysis results in this study, and provided the theoretical basis for the anti-inflammatory and analgesic mechanism and Q-marker research of Shiyifang Vinum. In the later stage of the experiment, we will determine the Q-marker through “spectrum effect generation” correlation experiments such as fingerprints, pharmacological activities, and blood components of Shiyifang Vinum, to reflect the internal relationship between the active components and the pharmacodynamic activities of the liquor, to continuously improve the anti-inflammatory and analgesic mechanism and Q-marker research of the liquor.

## Figures and Tables

**Table 1:** The medicinal materials and main chemical constituents of eleven prescriptions medicinal wine.

Herb name	Content of herb (mg/ml)	Main chemical components
Rhubarb	15	Chrysophanol, aloe emodin, beta-sitosterol, kaempferol, etc.
Safflower	10	Hydroxysafflor yellow A, luteolin, quercetin, beta-carotene, stigmasterol, baicalein, kaempferol, beta-sitosterol, etc.
Lycopus	20	Apigenin, beta-sitosterol, rosmarinic acid, etc.
Lignum sappan	10	Brazil hematoxylin, hematoxylin, isoliquiritigenin, purple riveting anthocyanin, etc.
Paris polyphylla	18	Paris polyphylla saponins, dioscin, etc.
Ground beetle	10	Beta-sitosterol, glycine, aspartic acid, etc.
Amber	15	Succinic acid, succinyl alcohol, etc.
Dragon's blood	15	Longxuesu A, longxuesu B, beta-sitosterol, liquiritigenin, terpineol, etc.
Notoginseng	12	Ginsenoside, quercetin, *β*-sitosterol, etc.
Pyritum	8	FeS_2_
Dipsacus	15	Saponin VI, *β*-sitosterol, loganin, etc.
Nux vomica	20	Brucine, strychnine, stigmasterol, etc.
Myrrh	20	Quercetin, *β*-sitosterol, ellagic acid, etc.

**Table 2:** Docking results of compounds and target proteins in Shiyifang Vinum.

Compound	Affinity (kJ·mol^−1^)							
	AKT1	MAPK1	MAPK14	PTGS2	TNF	PIK3CG	TP53	JUN
Beta-sitosterol	−59.83	−49.79	−46.44	−46.44	−36.82	−42.26	−35.56	−33.89
Ellagic acid	−41.42	−39.33	−32.22	−30.54	−43.93	−38.07	−29.71	−23.43
Aloe emodin	−42.26	−36.82	−32.64	−28.03	−41.84	−35.56	−28.03	−23.01
Aureusidin	−41.42	−36.82	−33.05	−33.05	−29.71	−37.24	−28.45	−25.52
Beta-carotene	−41.42	−35.15	−36.82	−39.33	−33.47	−35.98	−31.8	−28.45
Luteolin	−41.00	−37.66	−32.64	−34.73	−30.54	−38.49	−29.29	−25.10
Apigenin	−40.17	−35.98	−33.89	−33.89	−35.98	−35.15	−28.03	−24.69
Baicalein	−39.75	−37.24	−32.64	−34.73	−35.15	−36.40	−27.61	−25.10
Naringenin	−39.75	−35.56	−32.22	−34.31	−28.87	−35.98	−28.45	−24.27
Quercetin	−39.75	−38.49	−33.47	−33.89	−30.54	−37.66	−29.71	−25.10
Kaempferol	−38.91	−36.40	−33.05	−35.15	−28.87	−37.24	−27.61	−24.27
Isoliquiritigenin	−38.07	−31.38	−28.87	−29.71	−26.78	−27.61	−25.52	−23.01
Ginsenoside	−35.98	−31.80	−36.82	−34.73	−33.05	−36.40	−30.54	−30.96
Stigmasterol	−33.05	−33.05	−36.82	−33.89	−29.71	−35.56	−25.52	−28.87

## Data Availability

The data that support the findings of this study are available from the first author upon reasonable request.
